# Localization of a red fluorescence protein adsorbed on wild type and mutant spores of *Bacillus subtilis*

**DOI:** 10.1186/s12934-016-0551-2

**Published:** 2016-09-08

**Authors:** Giuliana Donadio, Mariamichela Lanzilli, Teja Sirec, Ezio Ricca, Rachele Isticato

**Affiliations:** 1 Department of Biology, Federico II University, via Cinthia-MSA, 80126 Naples, Italy; 2 School of Life Sciences, Gibbet Hill Campus, The University of Warwick, Coventry, CV4 7AL UK

**Keywords:** Display system, Heterologous protein delivery, Bacterial spore, Spore coat

## Abstract

**Background:**

Bacterial spores have been proposed as vehicles to display heterologous proteins for the development of mucosal vaccines, biocatalysts, bioremediation and diagnostic tools. Two approaches have been developed to display proteins on the spore surface: a recombinant approach, based on the construction of gene fusions between DNA molecules coding for a spore surface protein (carrier) and for the heterologous protein to be displayed (passenger); and a non-recombinant approach based on spore adsorption, a spontaneous interaction between negatively charged, hydrophobic spores and purified proteins. The molecular details of spore adsorption have not been fully clarified yet.

**Results:**

We used the monomeric Red Fluorescent Protein (mRFP) of the coral *Discosoma* sp. and *Bacillus subtilis* spores of a wild type and an isogenic mutant strain lacking the CotH protein to clarify the adsorption process. Mutant spores, characterized by a strongly altered coat, were more efficient than wild type spores in adsorbing mRFP but the interaction was less stable and mRFP could be in part released by raising the pH of the spore suspension. A collection of isogenic strains carrying GFP fused to proteins restricted in different compartments of the *B. subtilis* spore was used to localize adsorbed mRFP molecules. In wild type spores mRFP infiltrated through crust and outer coat, localized in the inner coat and was not surface exposed. In mutant spores mRFP was present in all surface layers, inner, outer coat and crust and was exposed on the spore surface.

**Conclusions:**

Our results indicate that different spores can be selected for different applications. Wild type spores are preferable when a very tight protein-spore interaction is needed, for example to develop reusable biocatalysts or bioremediation systems for field applications. *cotH* mutant spores are instead preferable when the heterologous protein has to be displayed on the spore surface or has to be released, as could be the case in mucosal delivery systems for antigens and drugs, respectively.

**Electronic supplementary material:**

The online version of this article (doi:10.1186/s12934-016-0551-2) contains supplementary material, which is available to authorized users.

## Background

Bacterial endospores (spores) are quiescent cells produced in response to harsh environments by Gram-positive bacteria mainly belonging to the *Bacillus* and *Clostridium* genera [1]. Spores can survive in their dormant state for long periods, resisting to a vast range of stresses such as high temperature, dehydration, absence of nutrients and presence of toxic chemicals. When the environmental conditions ameliorate, the spore germinates originating a vegetative cell able to grow and sporulate [2]. The ability of the spore to survive non-physiological conditions is, in part, due to its surface structures. In *Bacillus subtilis*, the model system for spore formers, the spore surface is organized in a multilayered coat and in a crust. The latter has been recently discovered and described as the outermost layer of the spore [2]. When observed by thin-section electron microscopy the spore coat appears formed by a lamellar inner coat and a more coarsely layered outer coat. The crust is only visible after a ruthenium red staining and appears as an amorphous layer surrounding the outer coat [2]. Coat and crust together are formed by at least 70 different proteins (Cot proteins) that spontaneously assemble into the multilayered structures, as recently reviewed [2]. *B. subtilis* spores are negatively charged [3, 4] and have a relative hydrophobicity, due in part to the glycosylation of some spore surface proteins [5].

The bacterial spore has been proposed as a platform to display heterologous proteins, with potential applications ranging from the development of mucosal vaccine to re-usable biocatalysts, diagnostic tools and bioremediation devices for field use [1, 6–8]. Various reasons support the use of the spore as a display system: (i) the remarkable and well documented resistance of the spore [2] that ensures high stability of the display system; (ii) the availability of genetic tools [9] that allows an easy manipulation; (iii) the safety record of several endospore-forming species [10, 11], that makes spores of those species ideal candidates also to deliver displayed molecules to mucosal surfaces [1, 8].

Two strategies have been so far developed to display heterologous proteins on the spore surface. A recombinant strategy based on the construction of gene fusions between the gene coding for a selected spore surface protein (carrier) and the heterologous DNA coding for the protein to be displayed was first developed to display an antigen, the C fragment of the tetanus toxin [12]. By this recombinant approach a variety of heterologous proteins have been displayed and recombinant spores proposed for several potential applications, as extensively reviewed [8]. More recently, a non-recombinant approach based on the spontaneous adsorption between purified spores and purified proteins has been also proposed [3]. Enzymes [13–15] and antigens [3, 16] have been efficiently displayed also by this approach and the system has been recently reviewed [17].

Spore adsorption is more efficient when the pH of the binding buffer is acidic (pH 4) and less efficient or totally inhibited at pH values of 7 or 10 [3, 15, 16]. A combination of electrostatic and hydrophobic interactions between spores and antigens has been suggested to drive the adsorption, which is not dependent on specific spore coat components [3, 17]. However, some mutant spores with severely altered spore surface were shown to interact more efficiently than isogenic wild type spores with the model enzyme beta-galactosidase [15]. In addition, heat-inactivated spores have been also shown to be able to efficiently display heterologous proteins [18, 19].

We used a fluorescent protein, the monomeric form of the Red Fluorescent Protein (mRFP) of the coral *Discosoma* sp. [20] and *B. subtilis* spores of a wild type and an isogenic mutant lacking the CotH protein [21] to analyze in more details the spore adsorption process. Spores lacking CotH have been previously shown to be more efficient than wild type spores in adsorbing the model enzyme beta-galactosidase [15] and to have a strongly altered coat: the inner coat is thinner than in wild type spores, the outer coat is much less electron-dense, and the two coat layers do not tightly adhere to each other [22]. These strong structural effects do not cause a strong phenotype in laboratory conditions and only a minor germination defect could be associated to the mutant spore [21]. CotH is a coat component with a regulatory role on other coat components [21], therefore spores lacking CotH also lack at least nine other proteins in the outer coat and crust [23]. Recently, it has been observed that the assembly of some CotH-dependent proteins, such as CotC, CotU, CotS and CotZ, is restored in spores lacking both CotH and CotG, another outer coat protein, and therefore that CotH counteracts CotG negative effects on spore coat assembly [24].

## Results and discussion

### mRFP of *Discosoma* sp. adsorbs to *B. subtilis* spores

**Fig. 1:**
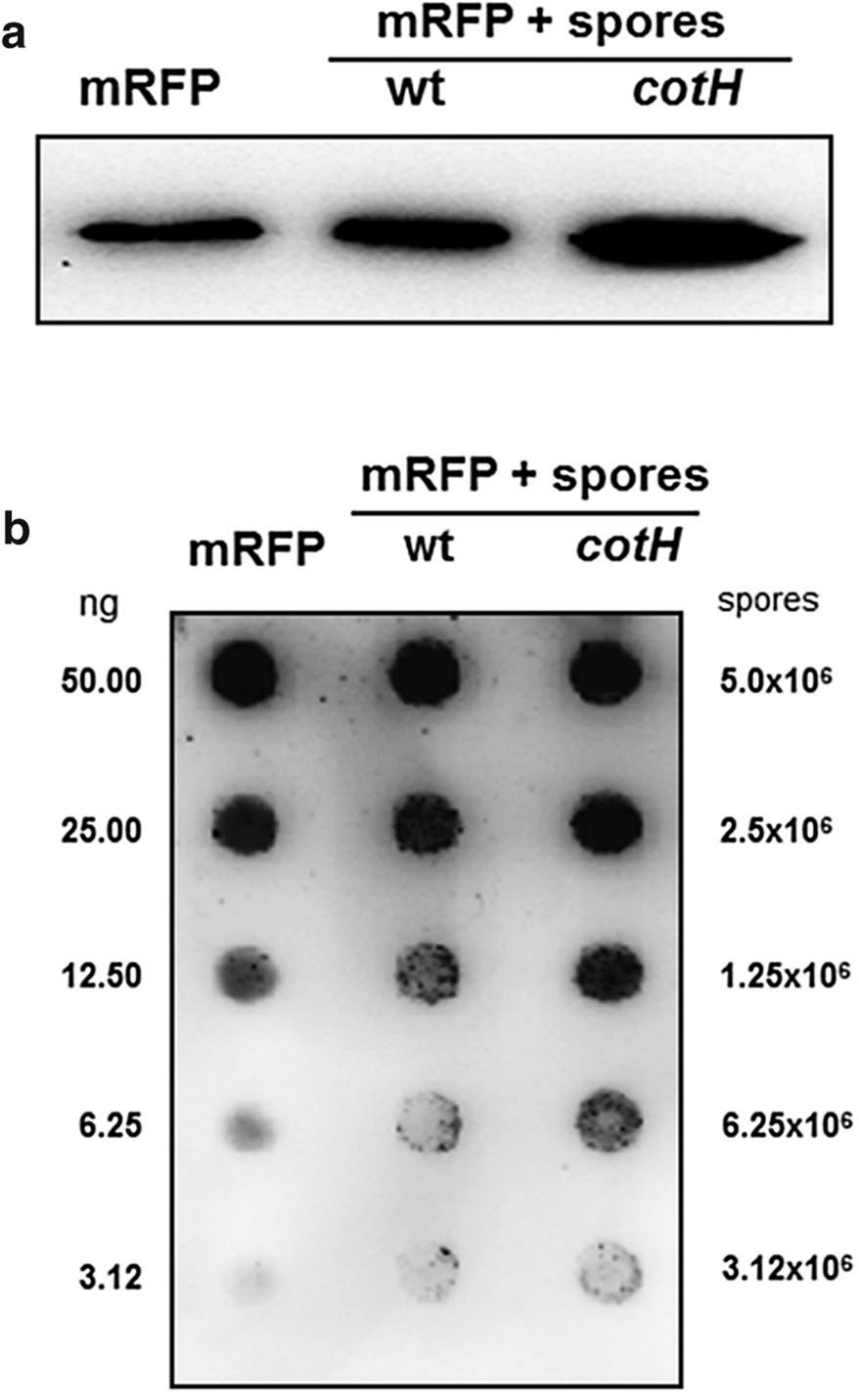
Western (**a**) and dot (**b**) blot of proteins extracted from wild type and *cotH* mutant spores after mRFP-spore adsorption. Purified recombinant mRFP is used as marker. In *panel b* the amount of purified recombinant mRFP (ng) and the number of spores present in each dilution are indicated. Immuno reactions were performed with anti-His primary antibody conjugated with horseradish peroxidase ("Methods'' section)

In an initial experiment 5 μg of mRFP of the coral *Discosoma* sp., over-expressed in *E. coli* and purified by affinity chromatography with Ni–Nta columns ("Methods" section), was incubated with 2.0 × 10^9^ purified spores of the *B. subtilis* strain PY79 [25] or of the isogenic strain ER209, lacking CotH [21]. The adsorption reactions were performed at pH 4.0, as previously described [15]. After the adsorption reaction spores were extensively washed, spore surface proteins were extracted as previously described [26] and analyzed by western blot with monoclonal anti-polyHistidine–peroxidase antibody (*Sigma*), able to recognize the his tagged N terminus of mRFP. As shown in Fig. 1a, mRFP was extracted from both wild type and *cotH* mutant spores, indicating that it was absorbed by both types of spores, retained during the washing steps and released by the extraction treatment. mRFP adsorption was extremely stable over time and the protein was still extracted by SDS–DTT treatment after 2 weeks of storage at room temperature of the adsorbed spores (Additional file 1: Figure S1).

Although the western blot of Fig. 1a is not quantitative, it suggested that *cotH* mutant spores released more mRFP than wild type spores. To confirm this suggestion by a quantitative approach we performed a dot blot experiment using purified mRFP as a standard. After the adsorption reaction spores were collected by centrifugation, serially diluted and analyzed by dot blotting (Fig. 1b). A densitometric analysis of the dot blot (Additional file 2: Table S1) showed that when 5 µg of mRFP were used in the adsorption reaction, wild type and mutant spores were able to adsorb, respectively, 3.40 µg (68 %) and 4.43 µg (88 %) of the total protein. (*P* = 0.00132). Therefore, indicating that mutant spores lacking CotH are more efficient than isogenic wild type spores in adsorbing mRFP.

**Fig. 2:**
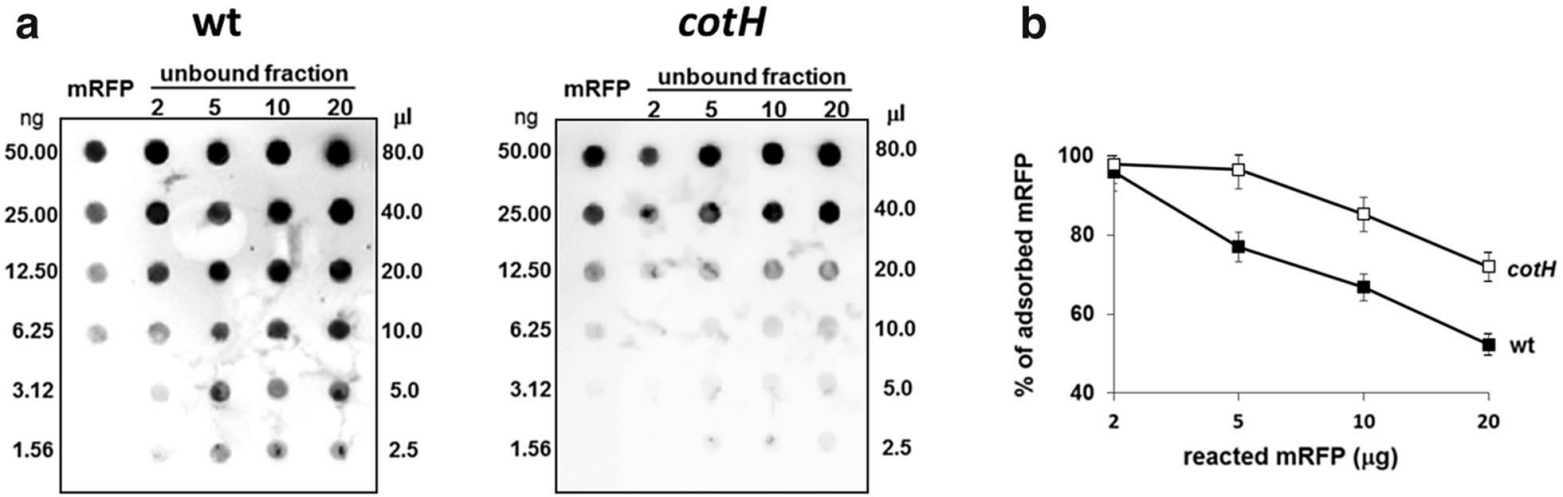
(**a**) Dot blot of serial dilutions of unbound mRFP after adsorption reactions with wild type and *cotH* mutant spores. The amount of purified recombinant mRFP (ng) and the volume of supernatant loaded are indicated. Immuno reactions were performed with anti-His primary antibody conjugated with the horseradish peroxidase (“Methods” section). **b** Efficiency of adsorption (%) of wild type and mutant spores (*black and white squares*, respectively) with various amounts of mRFP. The graph is designed on the base of the averages from densitometric analysis of three independent dot blots, reported in Additional file 2: Table S1. *Error bars* show the standard errors of the mean from the three experiments (*P* = 0.0025)

To analyze in more details the efficiency of adsorption, increasing amounts of mRFP (2, 5, 10 and 20 µg) were used in the reaction with the same amount of wild type or *cotH* mutant spores. In all cases the supernatant fraction of the adsorption reaction was serially diluted and analyzed by dot blotting with mRFP-recognizing anti-His antibody (Fig. 2a). Results of the densitometric analysis of the dot blots (Additional file 3: Table S2) are reported in Fig. 2b and indicate that with all amounts of mRFP tested, *cotH* mutant spores were more efficient than wild type spores in adsorbing the fluorescent protein.

### Adsorbed mRFP is tightly bound to spores

**Fig. 3:**
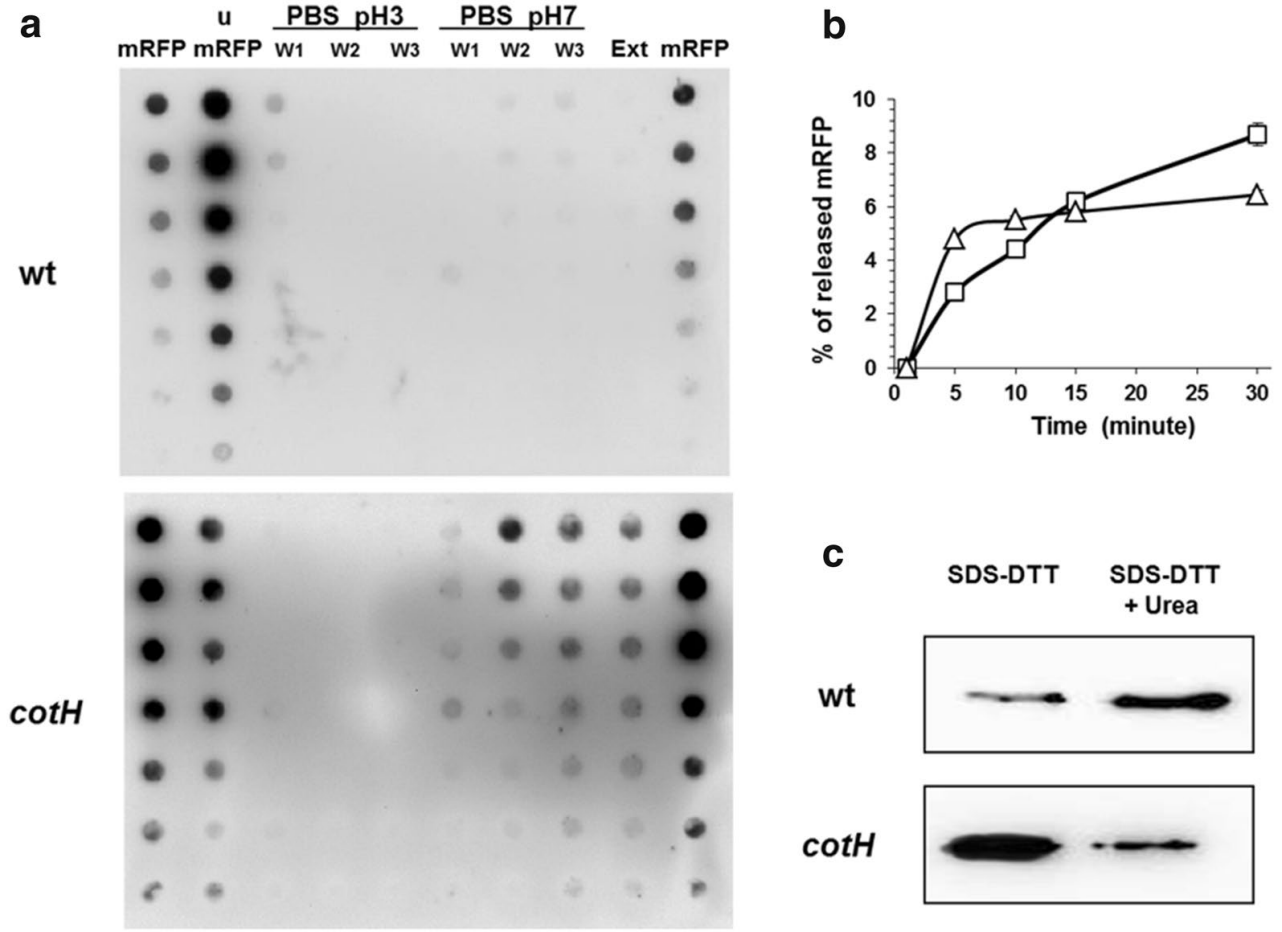
(**a**) *Dot blot* of released mRFP after three washes (W1, W2 and W3) with PBS buffer at pH 3 or pH 7 or after extraction with 1 M NaCl, 0.1 % Triton X-100 (Ext) (“Methods” section). Purified recombinant mRFP (mRFP) and unbound mRFP (u-mRFP) after the adsorption reaction were used as markers. **b** Kinetic of mRFP release based on the densitometric analysis (Additional file 4: Table S3) of mRFP released upon incubation either in PBS buffer pH 7.0 (*triangles*) or 1 M NaCl, 0.1 % Triton X-100 (*squares*). **c** Western blot of mRFP extracted by a SDS–DTT or after consecutive SDS–DTT and urea treatments of wild type and cotH mutant spores. Immuno reactions were performed with anti-His primary antibody anti-His primary antibody conjugated with the horseradish peroxidase (“Methods” section)

Upon adsorption to spores, mRFP is not easily released even after multiple washes. Spores adsorbed with mRFP were washed three times with 100 μl PBS buffer at pH 3.0 or pH 7.0 or extracted with 1 M NaCl, 0.1 % Triton X-100 [3]. With wild type spores no mRFP was released by any of these treatments, while with cotH mutant spores some mRFP was released by the washes at pH 7.0 and by the NaCl−Triton extraction (Fig. 3a). To evaluate the kinetic of mRFP-release adsorbed mutant spores were washed two times, resuspended in PBS pH 7.0 or 1 M NaCl, 0.1 % Triton X-100 for 5, 10, 15 or 30 min and the supernatant fractions analyzed by dot blot (not shown). A densitometric analysis of the dot blot (Additional file 4: Table S3) was performed and Fig. 3b reports the percentage of mRFP released by the two treatments at the different time points. The pH 7.0 buffer extracted about 5 % of the adsorbed mRFP within the first 5 min of incubation and did not extract more protein over time. The NaCl–Triton solution extracted about 3 % of mRFP in the first 5 min and the amount of extracted mRFP increased over time in an almost linear way reaching over 9 % of mRFP released after 30 min of treatment (Fig. 3b).

When spores were extracted with an SDS–DDT treatment, the standard protocol for extract spore coat proteins [26], mRFP was released by both wild type and mutant spores (Fig. 3c). The release of some mRFP after washes at pH 7.0 or 1 M NaCl, 0.1 % Triton X-100 treatment (Fig. 3a) and the high amount of mRFP released after SDS–DTT extraction by mutant spores may reflect the higher amount of mRFP adsorbed by mutant than wild type spores (Fig. 2). However, if spores previously extracted with SDS–DTT were re-extracted with Urea, a procedure used to completely remove the spore coat [27], additional mRFP molecules were released but, in this case, in higher amount from wild type than from mutant spores (Fig. 3c). Taken together the experiments of Figs. 2 and 3 suggest that wild type spores adsorb mRFP less efficiently but more tightly than *cotH* mutant spores.

### Localization of adsorbed mRFP on wild type and mutant spores

**Fig. 4:**
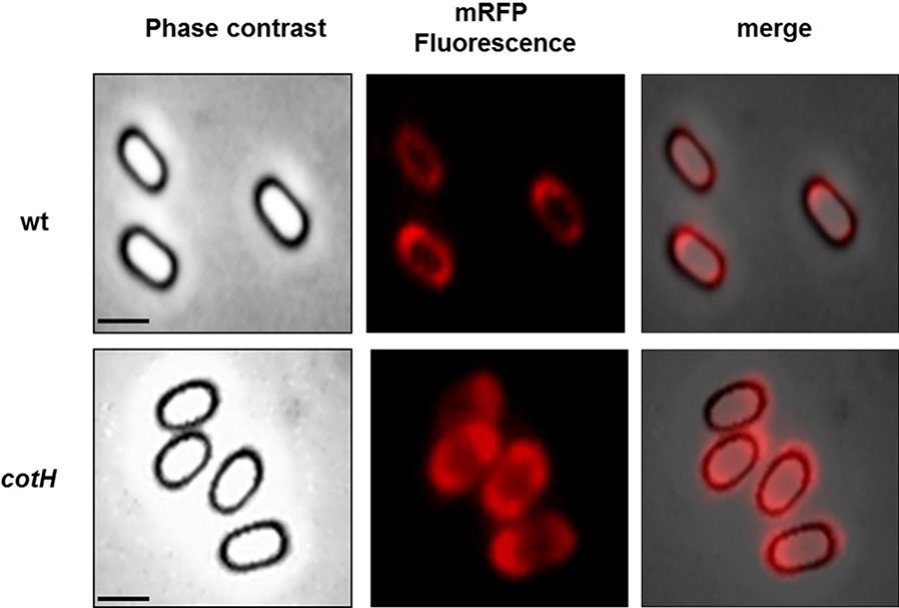
Fluorescence of mRFP upon adsorption to wild type and mutant spores. For each strain the same microscopy field was observed by phase contrast and fluorescence microscopy. Merge panels are reported. The exposure time was of 500 ms for both strains. *Scale bar* 1 μm

**Fig. 5:**
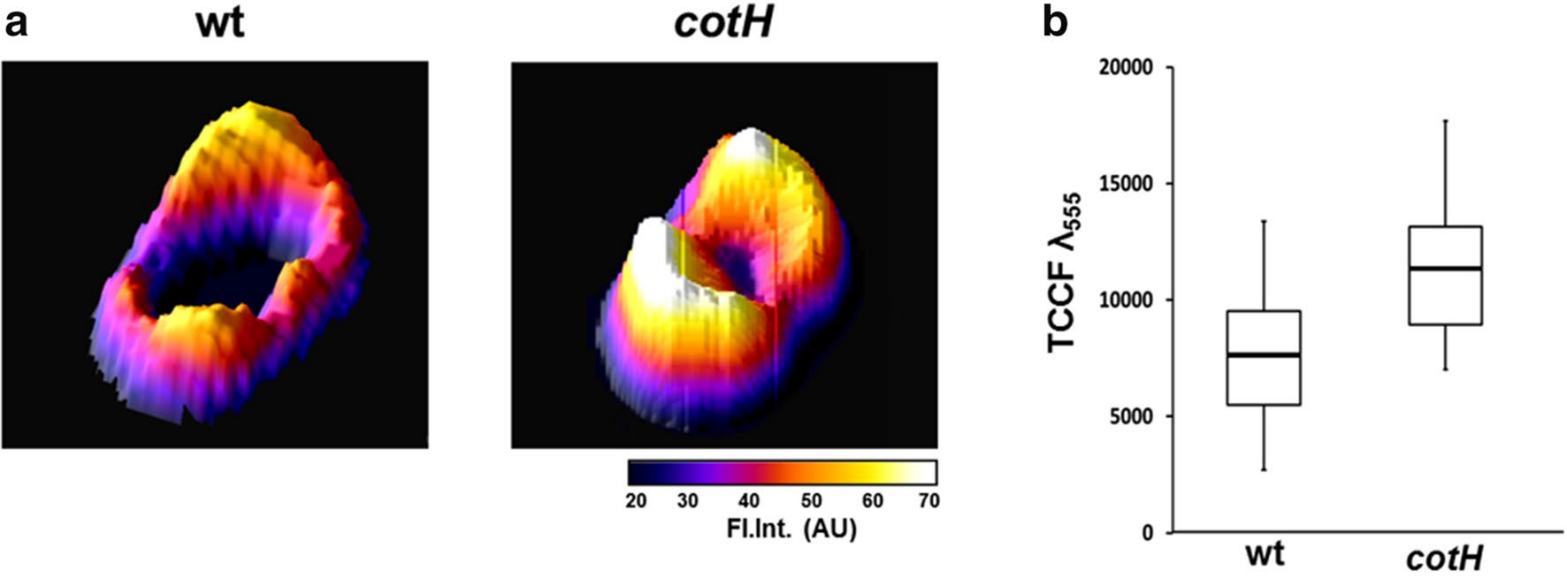
Fluorescence intensity profiles (Fl. Int.; scale in arbitrary units) of wild type and mutant spores adsorbed with mRFP. The profiles were generated from fluorescence microscopy images using the 3D Surface plotter function of Image J (http://imagej.nih.gov/ij/). **a** Representative fluorescence intensity profiles of a wild type (*left*) and *cotH* mutant spore (*right*). The fluorescence intensity is reported in arbitrary units. **b** Box plots displaying the total corrected cellular fluorescence (TCCF) for 80 different spores of each strain. Limits of each box represent the first and the third quartile (25 and 75 %) and the values outside the boxes represent the maximum and the minimum values. The line dividing the box indicates the median value for each strain. *P value* is less than 0.0001

To assess whether spore-adsorbed mRFP molecules retained their fluorescence properties and investigate their distribution around the spore we performed a fluorescence microscopy analysis. With both wild type and mutant spores red fluorescent signals were observed all around the spore (Fig. 4), and in agreement with results of Figs. 2 and 3, the fluorescence signal appeared stronger with mutant than with wild type spores (Fig. 4). In all cases the fluorescent signal was stronger at the spore poles, indicating that adsorbed mRFP molecules were not evenly distributed around the spore and accumulated at the poles (Fig. 4). We used Image J software (v1.48, NIH) to perform a quantitative fluorescence image analysis and the corrected spore fluorescence was calculated as described in the “Methods” section. The analysis of 80 spores of each strain indicated an average fluorescence intensity, in arbitrary units, of 7816 ± 2712 and of 11541 ± 2573 for wild type spores and mutant spores, respectively (Fig. 5, *P*  <  0.0001), confirming that *cotH* mutant spores adsorb more mRFP than wild type spores.

### Adsorbed mRFP is not exposed on the surface of wild type spores

**Fig. 6:**
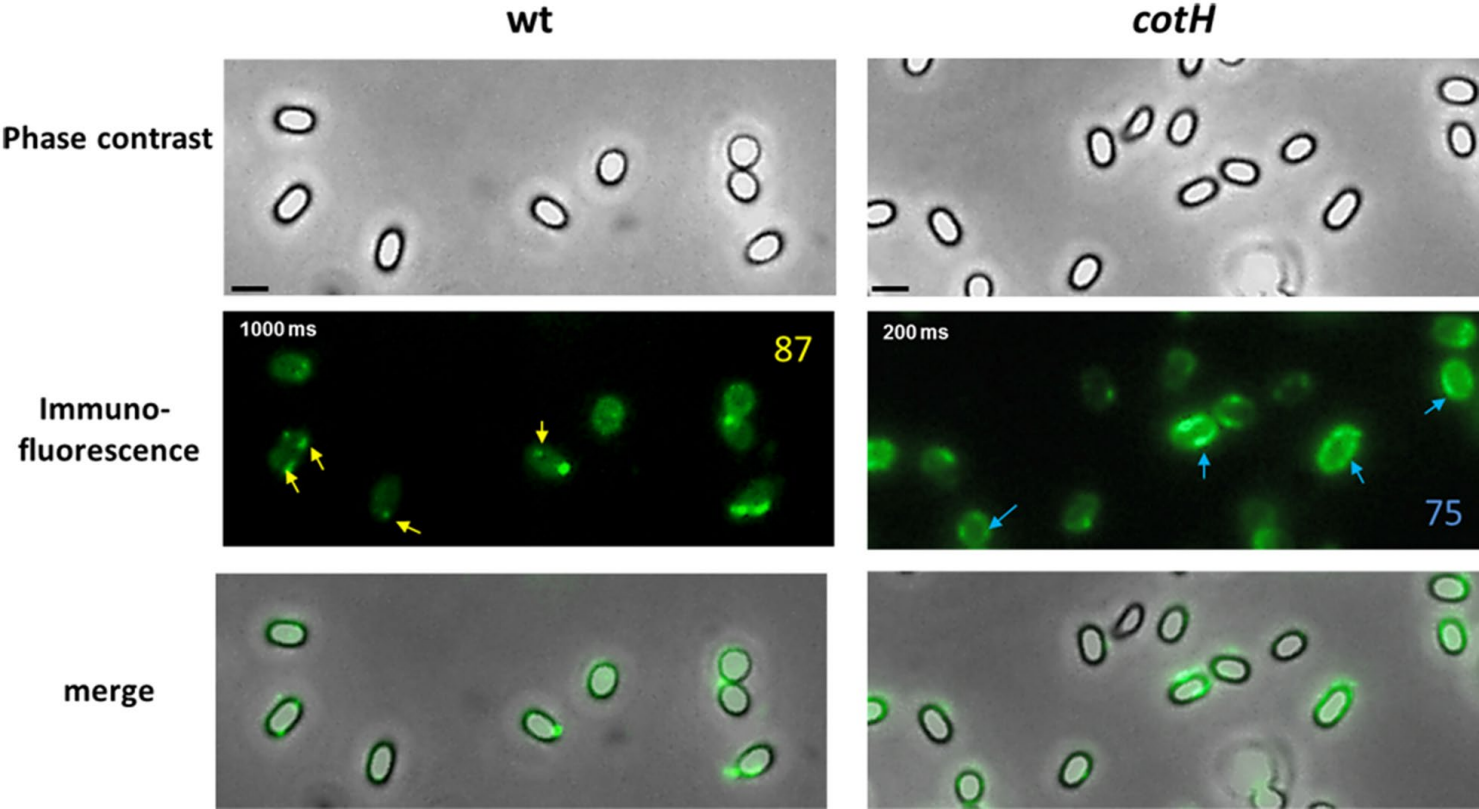
Immuno-fluorescence microscopy of wild type and mutant spores adsorbed with mRFP. Immuno reactions were performed with anti-His primary antibody and fluorescent anti-mouse secondary antibody conjugated with FITC (“Methods” section). For each strain the same microscopy field was observed by phase contrast and fluorescence microscopy. Merge panels are reported. The exposure time is indicated as ms. The* numbers in the panels* are the percentage of spores found with a similar mRFP-localization pattern in three different microscopy fields. *Scale bar* 1 μm

To assess whether adsorbed mRFP molecules were exposed on the spore surface we followed an immuno-fluorescence microscopy approach. Wild type and mutant spores adsorbed with mRFP were reacted with monoclonal mRFP-recognizing anti-His antibody, then with fluorescent anti-mouse secondary antibody (Santa Cruz Biotechnology, Inc.) and observed under the fluorescence microscope. While mRFP molecules adsorbed to mutant spores were accessible to the specific antibody all around the spore surface, mRFP molecules adsorbed to wild type spores were only poorly accessible to the antibody and only in specific spots mainly at the spore poles (Fig. 6). A fluorescent signal was observed with wild type spores only with an exposure time fivefold longer that used with mutant spores (Fig. 6). Results of Fig. 6, then indicate that mRFP molecules are mostly not accessible to the antibody upon adsorption on wild type spores. Since mRFP is abundantly present at the poles of wild type spores (Fig. 4) but it is only accessible to anti-mRFP antibody in few specific spots at the spore poles (Fig. 6) we hypothesize that either mRFP molecules bind wild type spores in an orientation that precludes the interaction with the antibody or that mRFP molecules infiltrate inside the spore coat layers of wild type spores.

### Adsorbed mRFP infiltrates in the inner coat of wild type spores

**Fig. 7:**
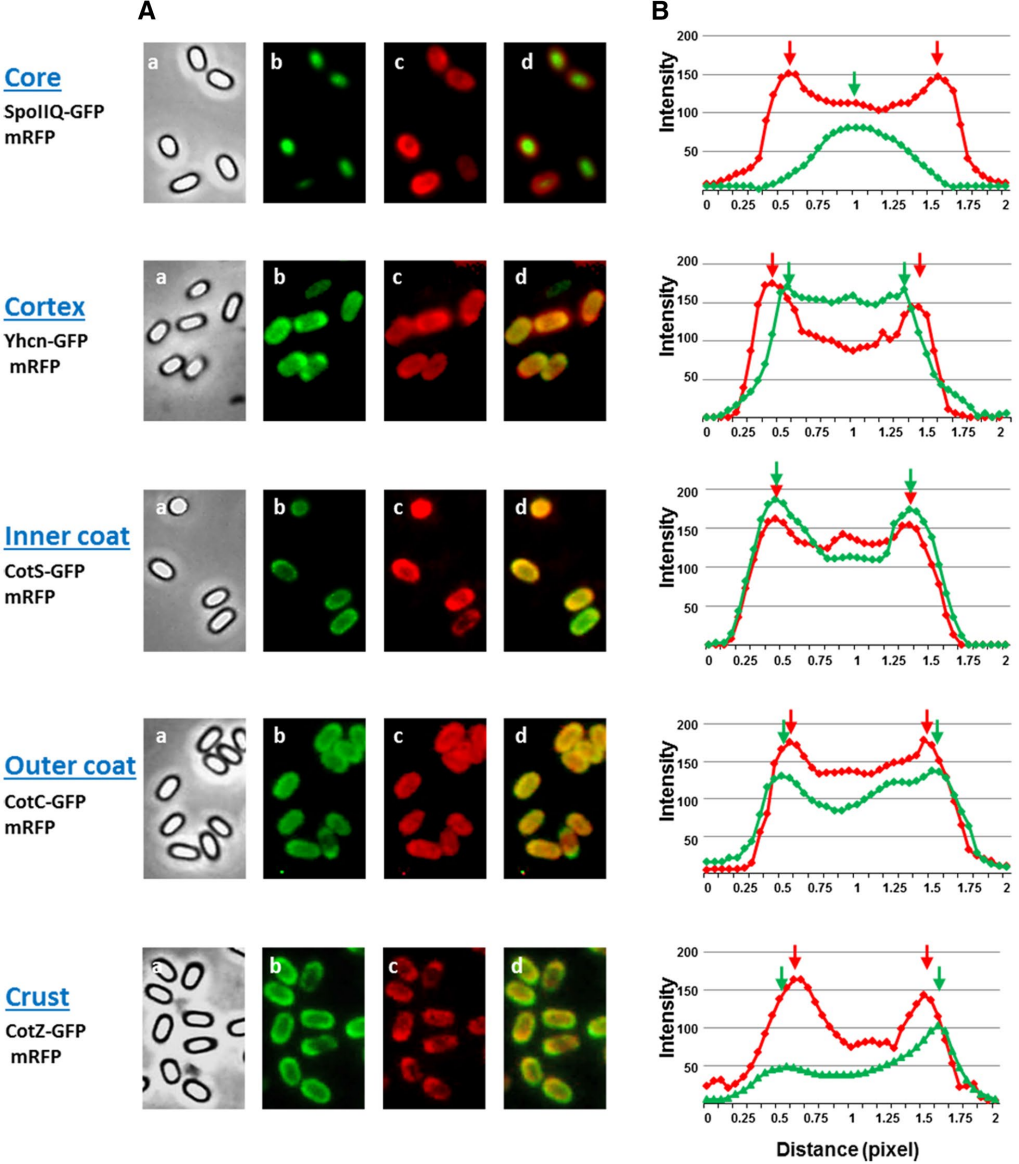
Fluorescence localization of coat protein-GFP fusions and adsorbed-mRFP (**A**) and plots of fluorescence intensities along the long axis of the spore (**B**). Spores of otherwise wild type *B. subtilis* strains carrying GFP fused to core, cortex, inner coat, outer coat or crust proteins were adsorbed with mRFP and observed by fluorescence microscopy. Phase-contrast images (*a*), GFP fluorescence images (*b*), mRFP fluorescence images (*c*) merged images (*d*) are shown

In order to verify whether mRFP molecules infiltrate inside the spore we used a fluorescence microscopy approach (“Methods” section). In particular we followed the red fluorescence of mRFP and the green fluorescence of GFP fused to *B. subtilis* proteins known to localize in the spore core (SpoIIQ [28]), cortex (YhcN [29, 30]), inner coat (CotS [31]), outer coat (CotC [30, 32]) or crust (CotZ [30]). Spores purified from isogenic strains carrying the *spoIIG::gfp, yhcN::gfp, cotS::gfp, cotC::gfp* or *cotZ::gfp* gene fusion were used to adsorb mRFP and observed by fluorescence microscopy (Fig. 7A). In order to determine the relative position of red and green signals quantitatively, more than 20 free spores for each strain were used to measure the intensity of GFP and RFP fluorescence along the spore axis by using image J software (“Methods” section). Averages of red and green fluorescence intensities of the various strains were plotted in Fig. 7B. With spores carrying the SpoIIQ–GFP (core) or the YhcN–GFP (cortex) fusion the green signal was internal with respect to the red one indicating that mRFP molecules were localized outside of the spore core (SpoIIQ) and of the cortex (YhcN). With spores carrying the CotS–GFP (inner coat) fusion the intensity peaks of green and red signals were coincident, indicating a co-localization of the two fluorescence signals. With spores carrying the CotC–GFP (outer coat) or the CotZ–GFP (crust) fusion, the intensity peaks of the green signals were slightly external to the red ones, indicating that in both cases mRFP molecules were inside the outer coat (CotC) and of the crust (CotZ) (Fig. 7). These results then indicate that mRFP molecules cross crust and outer coat layers and localize at the level of the inner coat of wild type spores, explaining why mRFP is not exposed on the surface of the spore (Fig. 6).

### Adsorbed mRFP localizes in all surface layers of *cotH* mutant spores

**Fig. 8:**
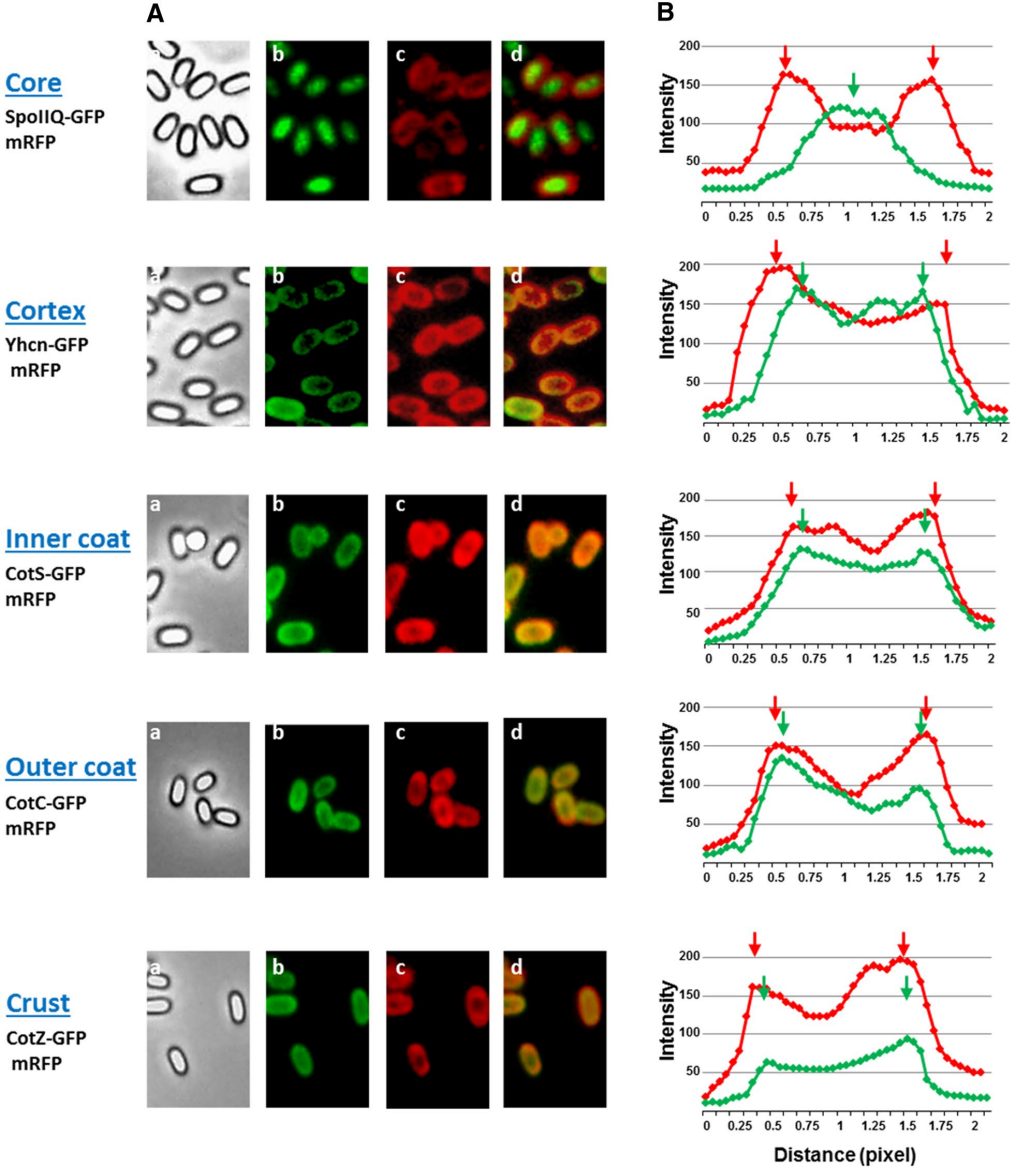
Fluorescence localization of coat protein-GFP fusions and adsorbed-RFP (**A**) and plots of fluorescence intensities along the long axis of the spore (**B**). Spores of *cotH* mutant strains carrying GFP fused to core, cortex or crust proteins and of *cotH cotG* mutant strains carrying GFP fused to inner and outer coat were adsorbed with mRFP and analyzed by fluorescence microscopy. Phase-contrast images (*a*), GFP fluorescence images (*b*), RFP fluorescence images (*c*) and merged images (*d*) are shown

The same approach was used to localize mRFP molecules inside mutant spores. Spores purified from isogenic strains carrying the *cotH* mutation and the *spoIIG::gfp, yhcN::gfp, cotS::gfp, cotC::gfp* or *cotZ::gfp* gene fusion were used to adsorb mRFP and observed by fluorescence microscopy (Fig. 8A). Since CotS (inner coat marker) and CotC (outer coat marker) fail to assemble within the coat of a *cotH* mutant [23, 33] but assemble normally in a strain lacking both CotH and CotG [24], we used a double mutant strain carrying null mutations in *cotH* and *cotG* genes for those two GFP fusions. As above, more than 20 free spores for each strain were used to measure the intensity of GFP and RFP fluorescence signals along the spore axis by using image J software (“Methods” section). Averages of red and green fluorescence intensities of the various strains were plotted in Fig. 8B. With spores carrying the SpoIIQ–GFP (core) or the YhcN–GFP (cortex) fusion the green signal was internal with respect to the red one indicating that mRFP molecules were localized outside of the spore core (SpoIIQ) and of the cortex (YhcN). With spores carrying the CotS–GFP (inner coat) and CotC–GFP (outer coat) fusion the red peaks were slightly outside the green ones and with the CotZ–GFP (crust) fusion the intensity peaks of green and red signals were coincident (Fig. 8). However, with mutant spores the red peaks did not appear sharp but rather broad suggesting that the red fluorescent molecules were not concentrated in a specific point but were diffused in a large space. Taken together these observations suggest a localization of mRFP molecules in all surface layers of *cotH* mutant spores that explains why mRFP is exposed on the surface of the spore (Fig. 6).

The experiments of Figs. 7 and 8 were performed using standard adsorption conditions: 5 μg of purified mRFP were incubated with 2.0 × 10^9^ purified spores for 1 h. To evaluate whether the amount of mRFP and/or the incubation time had an effect on mRFP localization we incubated 2.0 × 10^9^ wild type spores either with 50 μg of purified mRFP for 1 h or with 5 μg of mRFP for 4 h. In both cases mRFP localized in the inner coat of wild type spores as under standard conditions (Additional file 5: Figure S2), indicating that neither the amount of mRFP or the incubation time affected mRFP localization.

## Conclusions

**Fig. 9:**
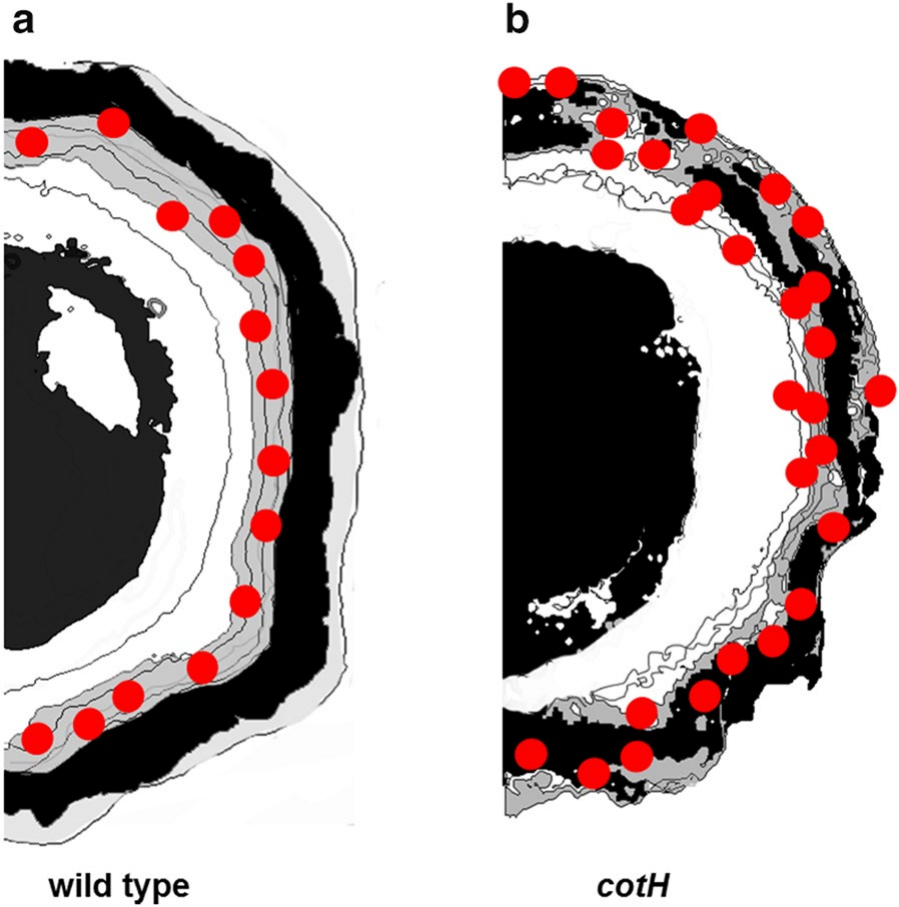
Cartoon model of mRFP localization in wild type (**a**) and *cotH* mutant (**b**) spores. Wild type and mutant spores are over imposed with *red dots* representing mRFP molecules

Wild type spores of *B. subtilis* adsorb and tightly bind mRFP that is not displayed on the spore surface. We propose that it infiltrates through crust and outer coat layers, localizes in the inner coat and appears more abundantly concentrated at the spore poles (Fig. 9a). The observation that mRFP crosses crust and outer coat indicates these structures as permeable to a 27 kDa protein. Permeability of the spore surface is not totally surprising since germinants (small molecules with molecular masses typically <200 Da) present in the environment have to cross the external layers of the spore to reach their receptors. In addition, studies conducted on *B. megaterium* suggested that the spore surface is permeable to the passage of molecules with masses somewhere between 2 and 40 kDa [34, 35].

Mutant spores lacking CotH are strongly defective: (i) their outer coat is diffuse, lacks the characteristic multilayered pattern, electron density, and a defined outer edge; (ii) the typical lamellar structure of the inner coat, in which three to five lamellae can usually be recognized, is reduced to one or two lamellae; (iii) the inner and outer coats are not tightly associated [22]; at least nine spore coat proteins are not present [23]. These mutant spores are more efficient than wild type spores in adsorbing mRFP that, although more abundant at the spore poles is present all around the spore and exposed on its surface. However, the interaction between mRFP and *cotH* spores is less stable than with wild type spores and mRFP is partially released by washes at pH 7.0 (Fig. 3). We propose that mRFP molecules adsorb better to *cotH* mutant than to wild type spores because they not only localize in the inner coat but also in the outer coat and in the crust (Fig. 9b).

The observation that neither the amount of heterologous protein or the time of incubation affect mRFP localization in wild type spores (Additional file 1: Figure S1) argue that in those spores crust and outer coat are compact structures that allow mRFP transit but not accumulation. In *cotH* spores the outer coat is rather diffuse [22] and allows mRFP to localize and accumulate.

These findings shed light on the mechanism of spore adsorption and indicate that different spores, wild type or mutant, can potentially have different applications. As a carrier of enzymes to be re-used several times a surface display of the enzyme may be not strictly required while a tight adhesion to the carrier could desirable making wild type spores preferable over the mutant spores. On the contrary, as a drug/vaccine delivery vehicle a surface display could be essential and the release of the drug/antigen in some cases an useful property, making mutant spores preferable over the wild type.

## Methods

### Bacterial strains and transformation

*Bacillus subtilis* strain PY79 strain [25] was used as a wild type. Strain ER220 (*cotH* null mutant [21] and all other mutant strains of *B. subtilis* used in this study were isogenic derivatives of PY79. *B. subtilis* strains used in this study are listed in Additional file 6: Table S4. Isolation of plasmid DNA, restriction digestion, ligation of DNA and transformation of *E. coli* competent cells were carried out by standard methods [36].

### Construction of *gfp* fusions

The coding sequence of *yhcN* (564 bp) and the promotor region of *spoIIQ* (500 bp) were PCR amplified using *B. subtilis* chromosomal DNA as a template and synthetic oligonucleotide pairs spoIIQ-F/spoIIQ-R and yhcN-F/yhcN-R (Additional file 7: Table S5) respectively to prime the reactions. Purified DNA fragments were cloned in frame to the 5′ end of the *gfp* gene [37]. Plasmids were used to transform competent cells of strain PY79 yielding strain RH2466 (*P*_*spoIIQ*_*::gfp*) and RH282 (*yhcN::gfp*). Chromosomal DNA of the two strains and of AZ573 (*cotZ::gfp*) was used to transform competent cells of isogenic strains RH220 (*cotH::spc*) (Additional file 6: Table S4), yielding strains RH285 (*P*_*spoIIQ*_*::gfp cotH::spc*) and RH284 (*yhcN::gfp cotH::spc*) and RH278 (*cotZ::gfp cotH::spc*). Chromosomal DNA of strains DS127 (*cotC::gfp*) was used to transform competent cells of isogenic strains AZ603 (*ΔcotG ΔcotH::neo*), yielding strains AZ636 (*cotC::gfp ΔcotG ΔcotH::neo*).

### Purification of spores and RFP

Sporulation of wild type and recombinant strains was induced by the exhaustion method. After 30 h of growth in Difco Sporulation medium at 37 °C with vigorous shaking [36] spores were collected, washed three times with distilled water and purified on a step gradient of 20–50 % of Gastrografin (Bayer) as described before [38, 39]. The number of purified spores obtained was measured by direct counting with a Bürker chamber under an optical microscope (Olympus BH-2 with 40× lens).

Competent cells of *E. coli* strain BL21(DE3) (Invitrogen) was transformed with the expression vector pRSET-A carrying an in-frame fusion of the 5′ end of the *rfp* coding region to six histidine codons under the transcriptional control of a T7 promoter (generous gift of Dr. A. Storlazzi). The new strain RH161 was grown for 18 h at 37 °C in 100 ml of autoinduction medium to express the heterologous protein [40]. The six-His-tagged RFP protein was purified under native conditions using a His-Trap column as recommended by the manufacturer (GE Healthcare Life Science). Purified protein was desalted using a PD10 column (GE Healthcare Life Science) to remove high NaCl and imidazole concentrations and mRFP purity analyzed by SDS-PAGE.

### Adsorption reaction

Unless otherwise specified 5 μg of purified recombinant mRFP were added to a suspension 2 × 10^9^ spores in 0.15 M PBS pH 4.0 at 25 °C in a final volume of 200 μl. After 1 h of incubation, the binding mixture was centrifuged (10 min at 13,000*g*) to fractionate pellet and supernatant. The pellet was resuspended in 0.15 M PBS at pH 4.0 to a final concentration of 2 × 10^5^ spores/μl and stored at 4 °C. The supernatant was stored at −20 °C.

### Western and dot-blot analysis

2 × 10^8^ mRFP-adsorbed spores were resuspended in 20 μl of spore coat extraction buffer [26], incubated at 68 °C for 1 h to solubilize spore coat proteins and loaded onto a 12 % SDS-PAGE gel. The proteins were then electro transferred to nitrocellulose filters (Amersham Pharmacia Biotech) and used for Western blot analysis as previously reported [39] using monoclonal mRFP-recognizing anti-His antibody (*Sigma*). A quantitative determination of the amount of mRFP was obtained by dot blot experiments analyzing serial dilutions of purified RFP, binding assay supernatant and RFP-adsorbed spores. Filters were then visualized by the ECL-prime (Amersham Pharmacia Biotech) method and subjected to densitometric analysis by Quantity One 1-D Analysis Software (Bio-Rad).

### Fluorescence and immunofluorescence microscopy

Post adsorption spores were resuspended in 50 μl of 1× PBS and 5 μl of the suspension placed on microscope slides and covered with a coverslip previously treated for 30 s with poly-l-lysine (*Sigma*). Immunofluorescence was performed as described by Isticato et al. [16], with the following modifications: 2.0 × 10^6^ RFP-adsorbed spores of wild type and *cotH* mutant spores were pretreated with 1 % bovine serum albumin (BSA)—1× PBS, pH 4.0 for 30 min prior to 2 h-incubation a 4 °C with the monoclonal anti-polyHistidine antibodies (mouse) (*Sigma*) diluted 1:20 in 1× PBS−1 % BSA. As a control of the specificity of this technique, non-adsorbed spores were directly treated with anti-His antibodies. After three washes, the samples were incubated with a 64-fold diluted anti-mouse secondary antibody conjugated with Fluorescein isothiocyanate, FITC (Santa Cruz Biotechnology, Inc.) and washed four times with PBS. Washed samples were resuspended in 20 μl of 1× PBS and 10 μl were analyzed. All samples were observed with an Olympus BX51 fluorescence microscope fitted with a 100× objective UPlanF1; U-MNG or U-MWIBBP cube-filters were used to detect the red and green fluorescence emission respectively. Images were captured using an Olympus DP70 digital camera equipped with Olympus U-CA Magnification Changer and processed with Image Analysis Software (Olympus) for minor adjustments of brightness, contrast and color balance and for creation of merged images of GFP and RFP [41]. Exposure times were in the range between 200 and 2000 ms for image capture of GFP fusions and for adsorbed RFP. Fluorescence intensities and the distance between two fluorescent peaks were measured using unadjusted merged images with Image J processing software (version 1.48, NIH) as previously describe by Imamura and collaborators [42]. One pixel corresponds to 1.18 nm in our detection system. Image J was also used to draw an outline around 80 spores for each strain, prior to area, integrated density and the mean fluorescence measurements being recorded, together with several adjacent background readings. The total corrected cellular fluorescence (TCCF) was calculated by subtracting (area of selected cell × mean fluorescence of background readings) from integrated density values. Minimum, maximum and mean value of TCCF were displayed as box-plots with 5–95 % confidence intervals [42]. Fluorescence intensity profiles were generated from the microscopy images using the 3D Surface plotter function of Image J as reported by Serrano and collaborators [43].

## Statistical analysis

Results from dot blot and fluorescence microscopy analysis are the averages from three independent experiments. Statistical significance was determined by the Student* t* test, and the significance level was set at *P* < 0.01.

## Additional files

10.1186/s12934-016-0551-2 Stability of spore adsorption over time. Purified suspensions of wt spores were mixed with purified recombinant mRFP (5μg) for 1 h at RT. Spores were centrifuged, washed two times, resuspended in PBS, pH3 and stored at RT. After 1, 2, 7 and 14 days, the adsorption mixtures were fractionated by centrifugation and the pellet fractions used for protein extraction by SDS-DTT treatment. Extracted proteins were fractionated on SDS-PAGE and analyzed by western blot. Immuno-reactions were performed with anti-His antibody conjugated with horseradish peroxidase.
10.1186/s12934-016-0551-2 Densitometric analysis of dot blot experiments reported in Fig 1 performed with the pellets of the adsorption reaction with wild type and cotH mutant spores.
10.1186/s12934-016-0551-2 Densitometric analysis of dot blot experiments reported in Fig 2 performed with the supernatants of the adsorption reaction performed with different amounts of mRFP and with wild type and cotH mutant spores.
10.1186/s12934-016-0551-2 Densitometric analysis of dot blot experiments with the supernatants of adsorption reaction and with washes after various time of incubation with 1× PBS pH7.0 or 1M NaCl–0.1% Triton 100.
10.1186/s12934-016-0551-2 Fluorescence microscopy analysis of spores of otherwise wild type B. subtilis strains carrying GFP fused to inner coat, outer coat or crust proteins adsorbed with 50 μg of purified mRFP for 1 h (A) or with 5 μg of purified mRFP for 4 h (B). For each strain the same microscopy fields was observed by phase contrast and fluorescence microscopy (red and green). Merge and overlay (red on green or viceversa) are also shown.
10.1186/s12934-016-0551-2 *Bacillus subtilis* strains.
10.1186/s12934-016-0551-2 List of oligonucleotides used to prime amplification reactions.

## Abbreviations

mRFP: monomeric Red Fluorescent Protein
RT: room temperature
GFP: green fluorescent protein
SDS-DDT: sodium dodecyl sulfate–dithiothreitol
NIH: National Institute of Health
PBS: phosphate saline buffer
TCCF: total corrected cellular fluorescence
