# Supplementary figures and images for: Localization of a red fluorescence protein adsorbed on wild type and mutant spores of Bacillus subtilis
Source: Microb Cell Fact. 2016 Sep 8;15(1):153. doi: 10.1186/s12934-016-0551-2 (PMC5016992; doi:10.1186/s12934-016-0551-2)

**mRFP**

**mRFP - spores**

---

**1**

**2**

**7**

**14**

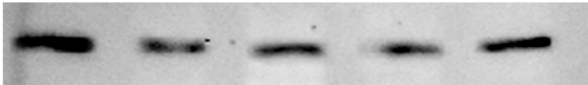

Supplement: Supplementary file 1 — 10.1186/s12934-016-0551-2 Stability of spore adsorption over time. Purified suspensions of wt spores were mixed with purified recombinant mRFP (5μg) for 1 h at RT. Spores were centrifuged, washed two times, resuspended in PBS, pH3 and stored at RT. After 1, 2, 7 and 14 days, the adsorption mixtures were fractionated by centrifugation and the pellet fractions used for protein extraction by SDS-DTT treatment. Extracted proteins were fractionated on SDS-PAGE and analyzed by western blot. Immuno-reactions were performed with anti-His antibody conjugated with horseradish peroxidase. [file 12934_2016_551_MOESM1_ESM.pdf]
