# Supplementary material for: Localization of a red fluorescence protein adsorbed on wild type and mutant spores of Bacillus subtilis
Source: Microb Cell Fact. 2016 Sep 8;15(1):153. doi: 10.1186/s12934-016-0551-2 (PMC5016992; doi:10.1186/s12934-016-0551-2)
Supplement: Supplementary file 2 — 10.1186/s12934-016-0551-2 Densitometric analysis of dot blot experiments reported in Fig 1 performed with the pellets of the adsorption reaction with wild type and cotH mutant spores. [file 12934_2016_551_MOESM2_ESM.pdf]

Additional Table 1.

Densitometric analysis of dot blot experiments with the pellets of the adsorption reaction with wild type and *cotH* mutant spores.

mRFP source	Amount of sample used	Density (OD/mm2) [a]	Amount of mRFP (ng) [b]	mRFP µg in 200 µl (% total) [b]
**Purified mRFP**	50.0 ng	74.35	NA	NA
	25.0 ng	42.31	NA	NA
	12.5 ng	17.80	NA	NA
	6.25 ng	6.89	NA	NA
**wt**	5.0×10^{6}	59.14	39.28	3.4 (68%)
	2.5×10^{6}	39.05	17.33	
	1.25×10^{6}	7.43	7.85	
cotH	5.0×10^{6}	72.25	48.5	4.43 (92.6%)
	2.5×10^{6}	58.75	26.1	
	1.25×10^{6}	115.52	10.9	

[a] Density measured by optical density (OD) per square millimeter and obtained by ChemiDocXRS apparatus with Quantity-One software (Bio-Rad).

[b]Calculated from signals (density OD/mm2) obtained with purified mRFP.
NA, not applicable.
