# Supplementary material for: Localization of a red fluorescence protein adsorbed on wild type and mutant spores of Bacillus subtilis
Source: Microb Cell Fact. 2016 Sep 8;15(1):153. doi: 10.1186/s12934-016-0551-2 (PMC5016992; doi:10.1186/s12934-016-0551-2)
Supplement: Supplementary file 3 — 10.1186/s12934-016-0551-2 Densitometric analysis of dot blot experiments reported in Fig 2 performed with the supernatants of the adsorption reaction performed with different amounts of mRFP and with wild type and cotH mutant spores. [file 12934_2016_551_MOESM3_ESM.pdf]

Additional Table 2.

Densitometric analysis of dot blot experiments with the supernatants of the adsorption reaction performed with different amounts of mRFP and with wild type and *cotH* mutant spores

mRFP source	Amount of sample used	Density (OD/mm2) [a]	Amount of mRFP (ng) [b]	mRFP µg in 200 µl (% total) [b]
**Purified mRFP**	100.0 ng	193.17	NA	NA
	50.0 ng	83.52	NA	NA
	25.0 ng	39.28	NA	NA
	12.5 ng	13.92	NA	NA
**wt 2**	80.0 µl	160.77	85.52	0.182 (9.12%)
	40.0 µl	60.83	35.33	
	20.0 µl	19.42	15.92	
***cotH* 2**	80.0 µl	114.15	57.30	0.107 (5.35%)
	40.0 µl	40.8	27.25	
**wt 5**	20.0 µl	128.77	69.23	0.57 (11.4%)
	10.0 µl	56.83	33.33	
***cotH* 5**	20.0 µl	42.35	26.30	0.32 (6,4%)
	10.0 µl	16.04	15.85	
**wt 10**	5.0 µl	157.99	83.52	2.96 (29.6%)
	2.5 µl	65.36	38.33	
	1.2 µl	43.01	27.12	
***cotH* 10**	20.0 µl	193.61	101.30	1.29 (12.9%)
	10.0 µl	120.52	65.25	
	5.0 µl	66.32	39.11	
**wt20**	2.5 µl	166.25	88.25	7.43 (37.15%)
	1.2 µl	75.55	44.09	
***cotH*20**	2.5 µl	60.78	35.78	2.96 (14.8%)
	1.25 µl	42.31	26.80	

[a] Density measured by optical density (OD) per square millimeter and obtained by ChemiDocXRS apparatus with Quantity-One software (Bio-Rad).

[b] Calculated from signals (density OD/mm2) obtained with purified mRFP.
NA, not applicable.
