# Supplementary material for: Localization of a red fluorescence protein adsorbed on wild type and mutant spores of Bacillus subtilis
Source: Microb Cell Fact. 2016 Sep 8;15(1):153. doi: 10.1186/s12934-016-0551-2 (PMC5016992; doi:10.1186/s12934-016-0551-2)
Supplement: Supplementary file 4 — 10.1186/s12934-016-0551-2 Densitometric analysis of dot blot experiments with the supernatants of adsorption reaction and with washes after various time of incubation with 1× PBS pH7.0 or 1M NaCl–0.1% Triton 100. [file 12934_2016_551_MOESM4_ESM.pdf]

Additional Table 3.

Densitometric analysis of dot blot experiments with the supernatants of adsorption reaction (5 x 10^8 of cotH mutant spores - 2 µg of mRFP) and of washes with 1x PBS pH7.0 or 1M NaCl-0.1% Triton 100.

mRFP source	Amount of sample used	Density (OD/mm2) [a]	Amount of mRFP (ng) [b]	mRFP µg in 200 µl (% total) [b]
**Purified mRFP**	25.0 ng	25.125	NA	NA
	12.50 ng	10.78.40	NA	NA
	6.25 ng	4.62	NA	NA
**Unbound mRFP**	40.0 µl	11.56	12.55	0.083 (4.3%)
	20.0 µl	6.89	7.78	
	10.0 µl	2.99	3.55	
**<u>1x PBS pH 7.0</u>**				
**5 min**	40.0 µl	20.75	19.23	0.092 (4.8%)
	20.0 µl	13.42	12.92	
**10 min**	20.0 µl	12.5	13.03	0.107 (5.6%)
	10.0 µl	7.2	7.25	
**15 min**	20.0 µl	11.77	12.83	0.111 (5.8%)
	10.0 µl	6.83	5.33	
**30 min**	20.0 µl	17.35	15.30	0.122 (6.4%)
	10.0 µl	6.04	5.85	
**1M NaCl-0.1% Triton**				
**5 min**	80.0 µl	18.5	20.03	0.053 (2.8%)
	40.0 µl	10.2	11.25	
**10 min**	40.0 µl	14.83	15.89	0.084 (4.4%)
	20.0 µl	8.02	8.78	
**15 min**	20.0 µl	12.25	13.01	0.115 (6.2%)
	10.0 µl	7.28	7.31	
**30 min**	20.0 µl	15.31	16.80	0.164 (8.6%)
	10.0 µl	6.59	7.58	

[a] Density measured by optical density (OD) per square millimeter and obtained by ChemiDocXRS apparatus with Quantity-One software (Bio-Rad).

[b] Calculated from signals (density OD/mm2) obtained with purified mRFP.
NA, not applicable.
