# Supplementary material for: Localization of a red fluorescence protein adsorbed on wild type and mutant spores of Bacillus subtilis
Source: Microb Cell Fact. 2016 Sep 8;15(1):153. doi: 10.1186/s12934-016-0551-2 (PMC5016992; doi:10.1186/s12934-016-0551-2)
Supplement: Supplementary file 5 — 10.1186/s12934-016-0551-2 Fluorescence microscopy analysis of spores of otherwise wild type B. subtilis strains carrying GFP fused to inner coat, outer coat or crust proteins adsorbed with 50 μg of purified mRFP for 1 h (A) or with 5 μg of purified mRFP for 4 h (B). For each strain the same microscopy fields was observed by phase contrast and fluorescence microscopy (red and green). Merge and overlay (red on green or viceversa) are also shown. [file 12934_2016_551_MOESM5_ESM.pdf]

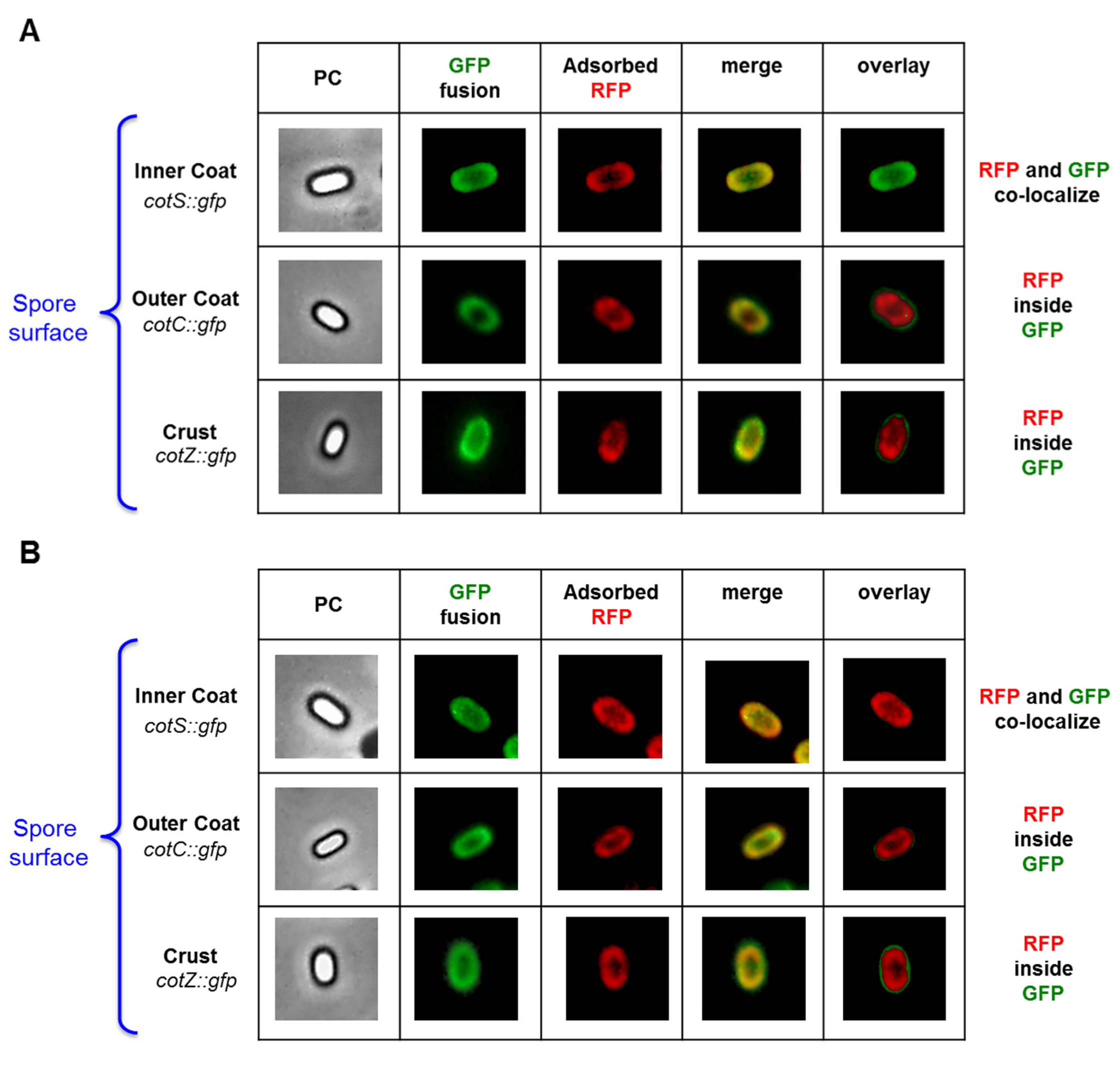
A
PC
GFP fusion
Adsorbed RFP
merge
overlay
Spore surface
Inner Coat
cotS::gfp
RFP and GFP co-localize
Outer Coat
cotC::gfp
RFP inside GFP
Crust
cotZ::gfp
RFP inside GFP
B
PC
GFP fusion
Adsorbed RFP
merge
overlay
Spore surface
Inner Coat
cotS::gfp
RFP and GFP co-localize
Outer Coat
cotC::gfp
RFP inside GFP
Crust
cotZ::gfp
RFP inside GFP
