# Supplementary material for: Localization of a red fluorescence protein adsorbed on wild type and mutant spores of Bacillus subtilis
Source: Microb Cell Fact. 2016 Sep 8;15(1):153. doi: 10.1186/s12934-016-0551-2 (PMC5016992; doi:10.1186/s12934-016-0551-2)
Supplement: Supplementary file 6 — 10.1186/s12934-016-0551-2 Bacillus subtilis strains. [file 12934_2016_551_MOESM6_ESM.pdf]

Additional Table 3.

***Bacillus subtilis* strains.**

**Strain**	**Genotype**	**Source**
PY79	*wild type*	[25]
ER220	*cotH::spc*	[21]
AZ573	*cotZ::gfp*	[5]
DS127	*cotC::gfp*	[35]
AZ644	*cotS::gfp*	[24]
AZ645	*cotS::gfp ΔcotG ΔcotH::neo*	[24]
RH2466	*pspoIIQ::gfp*	This study
RH285	*pSpoIIQ::gfp cotH::spc*	This study
RH282	*yhcN::gfp*	This study
RH284	*yhcN::gfp cotH::spc*	This study
AZ636	*cotC::gfp ΔcotG ΔcotH::neo*	This study
RH278	*cotZ::gfp cotH::spc*	This study
