# Supplementary material for: Localization of a red fluorescence protein adsorbed on wild type and mutant spores of Bacillus subtilis
Source: Microb Cell Fact. 2016 Sep 8;15(1):153. doi: 10.1186/s12934-016-0551-2 (PMC5016992; doi:10.1186/s12934-016-0551-2)
Supplement: Supplementary file 7 — 10.1186/s12934-016-0551-2 List of oligonucleotides used to prime amplification reactions. [file 12934_2016_551_MOESM7_ESM.pdf]

Additional Table 4.

List of oligonucleotides used to prime amplification reactions.

**Primer**	**Sequence** [a]	**Restriction site**
spoIIQ-F	ttcgaaATGAAGGCCATAAGTGAGCGGATGC	*Hin*dIII
spoIIQ-R	ctgcagTTCCTCTCTCATGTTTCATCACCTC	*Pst*I
yhcN-F	ttcgaaATGTTTGGAAAAAAACAAGTCCTTGCG	*Hin*dIII
yhcN-R	ctgcagAGCGTTAGGGAATACACGCTG	*Pst*I

[a] Not capital letters indicate restriction recognition sites.
